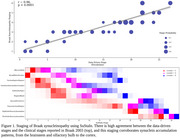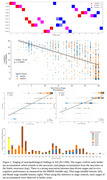# Data‐driven staging of postmortem neuropathology corroborates Braak staging and in‐vivo clinical and cognitive assessments in Alzheimer's’ and Parkinson's diseases

**DOI:** 10.1002/alz70855_105495

**Published:** 2025-12-24

**Authors:** Zaki Alasmar, Cécilia Tremblay, Geidy E Serrano, Thomas G Beach, Mahsa Dadar, Yashar Zeighami

**Affiliations:** ^1^ McGill University, Montreal, QC, Canada; ^2^ Banner Sun Health Research Institute, Phoenix, AZ, USA; ^3^ Civin Laboratory for Neuropathology, Banner Sun Health Research Institute, 10515 W Santa Fe Drive, Sun City, AZ 85351, Sun City, AZ, USA; ^4^ Douglas Mental Health University Institute, Montréal, QC, Canada; ^5^ Department of Psychiatry, McGill University, Montréal, QC, Canada

## Abstract

**Background:**

Identifying early biomarkers of neurodegenerative diseases is crucial for early disease intervention. Extending postmortem neuropathology insights to in‐vivo helps link impairment to biomarker accumulation. SuStaIn is a data‐driven approach that stratifies cross‐sectional neuropathology into longitudinal progression, enabling stage inference at group and individual levels. We aimed at testing whether SuStaIn can infer Braak synucleinopathy and Alzheimer's Disease (AD) amyloid plaque and neurofibrillary tangle progression.

**Methods:**

We applied OrdinalSuStaIn to Braak synucleinopathy gradings (2003; *N* = 110) to assess their progression. Similarly, we analyzed both plaque and tangle gradings from five brain regions in an AD cohort (*N* = 1300) and a control group (*N* = 972) using data from the Arizona Study of Aging and Neurodegenerative Disorders. To extend these findings in‐vivo, we evaluated the association between SuStaIn‐derived patient stages and cognitive performance.

**Results:**

SuStaIn successfully replicated Braak Lewy body progression, showing pathology initiation in the brainstem and olfactory bulb and later spread to limbic and cortical regions. These stages correlated strongly with the clinical Hoehn and Yahr's Parkinson's Disease (PD) staging (*r=*0.96, *p<*0.0001). For AD, SuStaIn captured postmortem plaque and tangle progression, starting with entorhinal tangles, progressing to hippocampal CA1 tangle deposition, and then to neocortical plaque and tangle accumulation. SuStaIn stages were strongly correlated with cognitive last MMSE scores (*r=*‐0.54, *p<*0.0001), Thal phases (*r=*0.80, *p<*0.0001), and Braak tangle stages (*r=*0.76, *p<*0.0001). advanced derived stages were associated with ApoE4 carriers in both data‐driven and pathological stages, while controls exhibited lower pathology levels consistent with early stages.

**Conclusions:**

Data‐driven approaches can elucidate neuropathological progression in PD and AD and capture additional nuances compared to previously used linear staging techniques. Here we first verify that as a staging tool, SuStaIn can corroborate Braak LB and tangle staging systems and provide a more quantitative model of deviations in individual cases. We further extend this approach in a large sample of postmortem AD neuropathology, showing that SuStaIn‐derived patient stages applied to both tangles and plaques were associated with their clinical disease stage and cognitive performance.